# The role of nutrition on thyroid health and disease: myths and facts

**DOI:** 10.1007/s40618-026-02852-0

**Published:** 2026-04-06

**Authors:** Rosaria Maddalena Ruggeri, Camilla Virili, Edoardo Mocini, Alfredo Campennì, Marco Centanni, Mario Rotondi, Salvatore Cannavò, Laura Croce, Silvia Migliaccio

**Affiliations:** 1 Department of Human Pathology of Adulthood and Childhood “G. Barresi”, Unit of Endocrinology, University of Messina, Messina, Italy; 2 Department of Medico-Surgical Sciences and Biotechnologies, “Sapienza” University of Rome, Latina, Italy; 3 Department of Theoretical and Applied Sciences, eCampus University, Novedrate, 22060 Italy; 4 Department of Biomedical and Dental Sciences and Morpho- Functional Imaging, University of Messina, Messina, Italy; 5 Department of Internal Medicine and Therapeutics, University of Pavia, Pavia, 27100 Italy; 6 Unit of Endocrinology and Metabolism, Laboratory for Endocrine Disruptors, Istituti Clinici Scientifici Maugeri IRCCS, Pavia, 27100 Italy; 7 Department of Experimental Medicine, University Sapienza of Rome, Rome, 00185 Italy

**Keywords:** Thyroid, Nutrition, Iodine, Selenium, Autoimmunity, Micronutrients, Mediterranean diet, Western diet

## Abstract

**Purpose:**

This narrative review examines the role of essential nutritional factors in thyroid function, moving beyond the traditional focus on iodine supplementation. The specific aim was to explore the association between food intake, specific nutrients, dietary patterns, and thyroid diseases, with particular emphasis on autoimmune mechanisms.

**Methods:**

A structured literature search was conducted using online databases to identify relevant studies published over the past two decades. Articles investigating the relationship between nutrients, dietary patterns, thyroid function, and autoimmune thyroid disorders were selected and analyzed.

**Results:**

Evidence confirms the crucial role of iodine and selenium in thyroid hormone synthesis and redox balance. Iron and zinc support enzymatic activity and endocrine stability. Vitamin D has emerged as a potential immunoregulatory factor in autoimmune thyroid diseases. Additionally, certain macronutrients and bioactive compounds, including polyphenols and omega-3 fatty acids, demonstrate beneficial effects due to their anti-inflammatory, antioxidant, and immunomodulatory properties. Adherence to the Mediterranean diet has been associated with favorable thyroid outcomes. In contrast, Western-style dietary patterns high in fat, calories, and red meat may negatively affect thyroid health, while gluten-free or lactose-free regimens do not appear to provide significant benefits.

**Conclusions:**

Evidence-based nutritional strategies represent valuable adjunctive approaches in the prevention and clinical management of thyroid disorders. Adherence to a Mediterranean dietary pattern and reduced meat consumption may improve thyroid function and help protect against autoimmune diseases.

## Introduction

Lifestyle and eating habits have changed significantly over the years, driven by shifts from traditional whole foods to a higher consumption of processed foods, increased convenience and snacking, along with the influence of technological advancements and globalization on food availability and consumption patterns [1–3]. New eating habits emerged, ranging from the widespread diffusion of the western-style diet, especially among younger individuals and low-income classes, on the one hand, to the rise of plant-based diets on the opposite [3–7]. In parallel, it has become evident how lifestyle and food habits significantly impact human health by influencing chronic disease risk, energy levels, and overall well-being [8]. In developed countries, excessive calorie intake and frequent consumption of “unhealthy foods,” associated with a sedentary lifestyle, have caused an increase in the prevalence of obesity, which in turn predisposes to several non-communicable diseases, such as cardiovascular disease, diabetes, hypertension, and chronic kidney disease [9]. Moreover, strong evidence links poor nutrition and obesity to the rising prevalence of chronic inflammatory and immune-mediated disorders, including autoimmune diseases [10]. In parallel, awareness of the role of nutrition, has grown significantly, extending across broader segments of the population and encompassing an ever-wider range of diseases, including thyroid disorders. As the prevalence of thyroid diseases is rising in the general population, particularly the autoimmune ones and among women [11, 12], expanding research has addressed the influence of dietary habits on thyroid health via micronutrients, the gut microbiota, and overall dietary patterns [13–16].

Not surprisingly, patients are increasingly seeking information on the role of diet in the treatment and prevention of thyroid diseases in daily clinical practice. Furthermore, the widespread use of the internet, and particularly the social media, has led to the spread of misinformation and incorrect nutritional advice, amplifying patients’ doubts and uncertainties. Interestingly, nutritional approaches, such as sugar-free diets or gluten-free, or “paleo diet” protocols, promoted for thyroid health, are gaining popularity, despite lacking solid scientific evidence. Lastly, iodized salt is often replaced with “natural” salts¸ that do not provide iodine intake.

As interest in dietary patterns has grown in the setting of thyroid disorders, the importance of tailored educational interventions has emerged, and our understanding of the link between diet and thyroid is rapidly expanding beyond the scope of iodine nutrition. Iodine, an essential micronutrient for the synthesis of thyroid hormones, is certainly the main element that links nutrition to the thyroid, but numerous other nutrients and dietary factors are fundamental for the health of the gland and must be taken into consideration in the prevention and treatment of thyroid diseases. The aim of this review is to examine the nutritional factors that are essential for correct thyroid function and to explore the correlations between intake of different foods, dietary patterns and thyroid diseases, with a special focus on autoimmunity.

## Materials and methods

An extensive literature search was carried out independently on online databases (MEDLINE via PubMed, ISI Web of Science, and Scopus), by four reviewers (RMR, EM, LC, and CV) independently of each other, to identify relevant studies published between January 2005 and June 2025. The following keywords and MESH terms were used: “thyroid diseases”, “thyroid autoimmunity”, AND “Mediterranean diet” OR “Western diet” OR “gluten-free diet” OR “lactose-free diet” OR “vegetarianism” OR “Low carbohydrate diet” OR “autoimmune protocol”. We also searched literature for associations between individual dietary components (iodine, selenium, ω-3 PUFAs, polyphenols, vitamins and micronutrients) and thyroid health and disease. Articles pertinent to the aim of the review were selected based on the relevance of the title and abstract in the topic by four authors (R.M.R., E.M., L.C., and C.V.) and then were critically evaluated by all authors. Articles written in the English language and published in peer-reviewed journals were included in the present review. Papers written in languages other than English were excluded.

### Micronutrients

#### Iodine

**Fig. 1:**
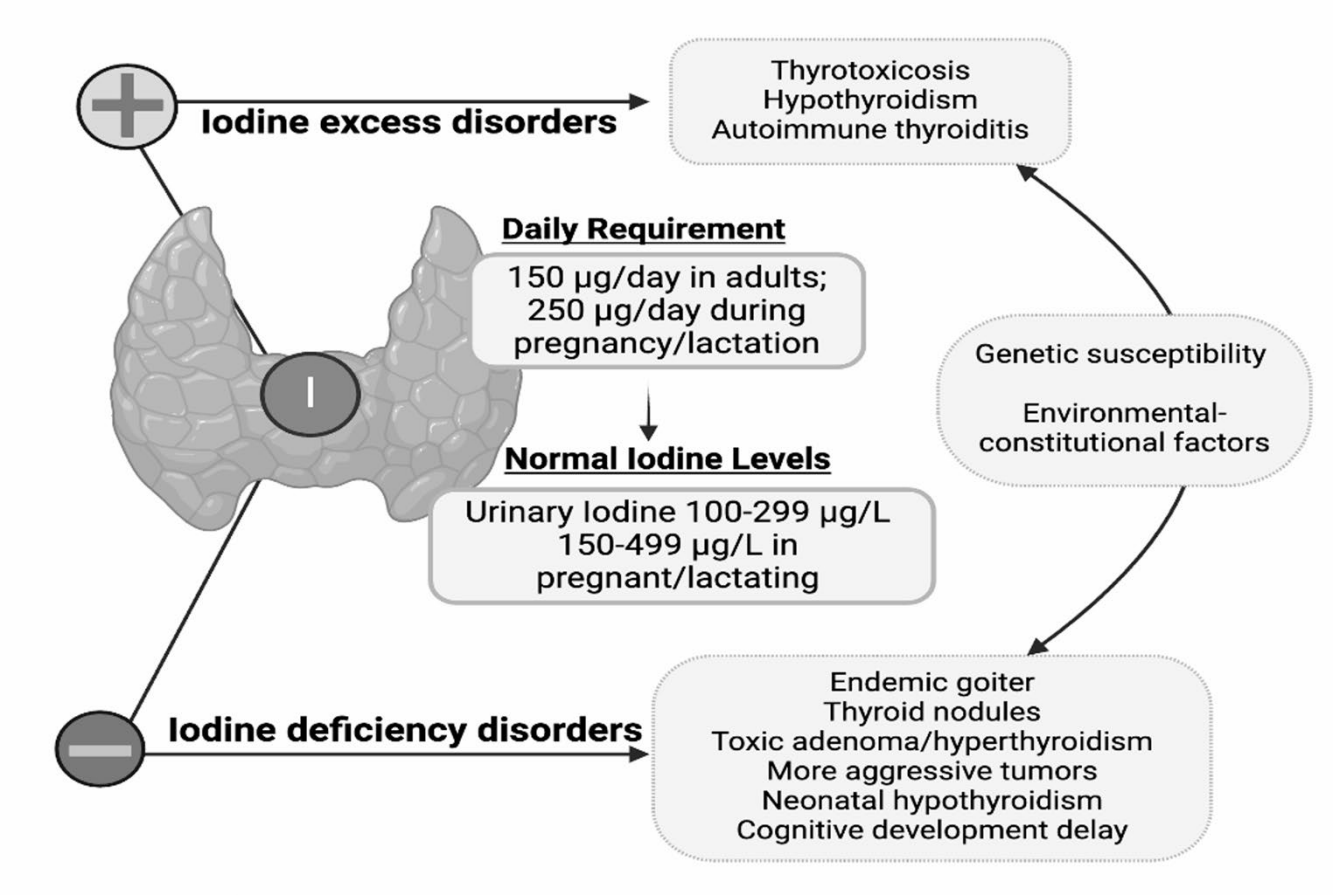
The U-shaped relationship between iodine intake and thyroid disorders. Both iodine deficiency and excess are associated with impaired thyroid function. Inadequate intake leads to goiter, nodular disease, and hypothyroidism, while excessive intake may precipitate hyperthyroidism or autoimmune thyroiditis in genetically susceptible individuals. Optimal iodine intake, 150 µg/day in adults and 250 µg/day during pregnancy and lactation, corresponds to urinary iodine concentrations of 100–299 µg/L and 150–499 µg/L, respectively

Iodine is an essential trace element, which plays a crucial role for thyroid hormones synthesis. Indeed, Iodine deficiency impairs normal thyroid function and is associated with a spectrum of thyroid disorders, the severity of which varies depending on the degree of deficit and the individual’s age [17, 18]. Iodine deficiency disorders (IDD) are entirely preventable by ensuring an adequate daily intake of iodine [17, 18] (Fig. 1). According to the World Health Organization (WHO), the recommended daily allowance (RDA) of iodine is 150 µg for adults and 250 µg for pregnant and lactating women, and iodine intake is assessed using median urinary iodine concentration (UIC) in the general population (not at individual levels) [19]. An adequate iodine intake is indicated by a median UIC of 100–199 µg/L in children and non-pregnant adults, and 150–249 µg/L in pregnant women. Values below these ranges suggest ID, with progressively lower levels reflecting greater severity (mild, UIC < 100 mg/L; moderate, UIC < 50 mg/L; severe, UIC < 20 mg/L) [19]. A Tolerable Upper intake Level (TUL) for iodine is established at 1100 µg/day for healthy subjects [20]. In accordance with the WHO criteria, the European Food Safety Authority (EFSA) has set the average requirement (AR) of iodine at 150 µg/day for adults, 70–130 µg/day for infants aged 7–11 months and children, and 200 µg/day for pregnant women. In addition, a Tolerable Upper intake Level (TUL) of 600 µg/day has been proposed for adults, including pregnant and lactating women [21]. An even lower TUL for iodine has been proposed by the American Thyroid Association (ATA), that advises against exceeding 500 µg per day of iodine, particularly in children, the elderly and during pregnancy [22]. Also, individuals with pre-existing thyroid disorders are considered more susceptible to potential adverse effects of excess iodine (either hypo or hyperthyroidism) [5, 22]. Indeed, a U-shaped relationship links iodine status to thyroid disorders, as both deficiency and excess can be detrimental (Fig. 1). Given the narrow range between inadequate and excessive intake, even minor variations may substantially affect the prevalence of thyroid dysfunction [17]. On the one hand, ID represents a major cause of hypothyroidism worldwide, and it also favors the development of goiter and thyroid nodules [6, 17, 23]. On the other hand, an excessive iodine intake, due to inappropriate supplementation or excess intake of iodine-rich food, increases the risk for hyperthyroidism/thyrotoxicosis, hypothyroidism and autoimmunity [7, 17, 24]. In addition, iodine supplementation in iodine-deficient areas has been associated with an increased risk of thyrotoxicosis, supporting the U-shaped relationship between iodine status and thyroid disorders [17, 18].

Notably, while excess iodine is well known to trigger thyroid autoimmunity in susceptible individuals, evidence from the literature is reassuring that an adequate iodine status (defined as a UIC between 100 µg/L and 299 µg/L) is safe in this regard [25, 26]. Worthy of mention, a rise in the prevalence of subclinical autoimmune hypothyroidism and thyroid autoantibodies has been reported after iodine supplementation in iodine-deficient countries, likely in relation to enhanced immunogenicity of thyroglobulin [27]. However, such a reported increase in autoimmunity after iodine supplementation appears to be largely transient and not associated with a long-term increase in thyroid dysfunction in most studies [7, 25, 26, 28]. Moreover, also iodine deficiency has been associated with increased autoimmunity [7, 25, 26], Overall, the benefits of iodine prophylaxis largely outweighing the risks, and a possible increase in thyroid autoimmunity should not represent a limitation to promoting iodine supplementation in the general population [25, 26, 29].

Iodine is naturally present in some foods, mainly in the form of iodide salt. Good dietary sources of iodine are represented by seafood, including marine fish, shellfish and seaweed [30, 31]. The iodine content of commercially available seaweeds may vary greatly, making it difficult to estimate an accurate average value, and some seaweed may largely exceed the TUL, exposing to iodine excess [32]. For this reason, uncontrolled consumption of seaweeds (such as macroalgae, nori and kelp), should be discouraged [33, 34]. Poultry, eggs, milk and dairy products also contain iodine, in a variable amount depending on animal feeding and hygienic practices [35]. Anyway, milk represents a relevant source of iodine, mainly in children, whereas plant-based beverages used as milk substitutes, such as soy and almond beverages, contain relatively small amounts of iodine [36]. Iodine is also present in some plant-based foods, like grains and cereals, with a variable content depending on iodine level in the soil where the food grows, which varies widely among countries, and on fertilizer use and irrigation practices; similarly, fruit and vegetables are a poor source of iodine [30, 31]. However, fortified plant-derived foods are available (i.e., certain types of bread and cereals, some vegetables), as well as milk infant formulas [31]. Moreover, cooking methods can reduce iodine content in foods due to its volatile nature, largely varying by cooking methods and food sources [37]. In contrast, the bioavailability of iodine increases after most cooking methods, though the overall amount absorbed may still be lower if the total iodine content is drastically reduced [37].

Therefore, dietary iodine intake, primarily dependent on fish consumption, is frequently below recommended levels in a large part of the population, and supplementation is necessary. The most reliable and cost-effective way to ensure adequate iodine intake for the general population is to use iodized salt, a common table salt enriched with iodine (30 mg for each kg of salt in Italy). The WHO recommends salt iodization as the preferred strategy to eradicate IDD [19], and several countries have adopted salt-iodization programs, successfully preventing IDDs and improving nutritional iodine status at a population level [38, 39]. The use of iodized salt is safe and without contraindications for the general population, including those with thyroid diseases, since it does not lead to excessive iodine intake. Recent evidence suggests that, even among hyperthyroid patients, maintaining an adequate dietary iodine intake improves therapeutic outcomes, enhancing response to treatment, reducing recurrence, increasing remission rates, and providing better control of thyrotoxicosis compared with inadequate or excessive iodine intake [40–42].

Moreover, while supporting thyroid function, an intake of iodized salt not exceeding 5 g per day does not increase the risk of hypertension and related cardiovascular diseases (CVD), and is consistent with CVD prevention strategies avoiding excessive sodium intake. Indeed, the major scientific societies [43–45] and the WHO 2023 statement [46] recommend that sodium intake should be limited to < 2 g per day (equivalent to < 5 g salt per day of added salt) in the general population as well as in hypertensive patients. Consequently, this should not be regarded as an impediment to the promotion of iodine supplementation within the general population; rather, the WHO recommends integrating salt/sodium reduction strategies with parallel salt iodization programs [46]. A varied and balanced diet, together with the regular use of iodized salt (no more than 5 g/day), ensures adequate iodine status without exposing to the risk of excessive iodine or sodium intake.

Noteworthy, a widespread reduction in salt intake on the one hand, and the frequent replacement of iodized salt by so-called “whole” salts (such as Himalayan pink salt), perceived as healthier alternatives, on the other hand, has contributed to the re-emergence of iodine deficiency in industrialized countries, with adverse health consequences, especially in children [47–49]. Specialty salts, such as sea salt, kosher salt, Himalayan salt, to mention a few, provide virtually no iodine [30]. Similarly, diets excluding animal products are becoming more popular worldwide, and if not properly planned and well balanced do not provide enough iodine, often requiring supplementation [34]. In particular, vegans, that excludes animal foods at all, might be at risk of both iodine deficiency (due to lack of animal products) and excess (due to consumption of vegan alternatives like seaweed) and related thyroid effects (Fig. 1) [34, 49]. Pregnant women, vegans, individuals with certain food allergies, those with lactose intolerance, and others who consume little or no dairy, fish, or iodized salt may be at risk of inadequate iodine intake. In such cases, iodine nutrition should be improved through pharmacological supplementation containing iodine in appropriate amounts. A titrated dose of 150 µg/day is generally recommended [34, 50, 51]. Many dietary supplements provide iodine as potassium iodide or from algae sources (mainly kelp), with a titrated dose within the range of 150–225 µg/day. However, several over-the-counter dietary and herbal products, marketed to support thyroid health and to lose weight, contain excessively high amounts of iodine, sometimes above the TUL, and their use should be discouraged, according to the ATA recommendations [22, 34, 52].

Finally, the lack of other micronutrients, as well as consumption of foods containing goitrogens, can exacerbate iodine deficiency. Foods high in goitrogens include soy, cassava, and cruciferous vegetables (e.g., cabbage, broccoli, and cauliflower). However, despite these substances are able to interfere with iodine uptake in the thyroid, the consumption of reasonable amounts of foods containing goitrogens is not a concern for people who have adequate iodine intakes and eat a variety of foods, and there are no contraindications to their consumption, as detailed below [53].

As concerns other micronutrients, deficiencies of selenium and/or iron or zinc would increase the detrimental effect of iodine deficiency on thyroid function, and/or impair antioxidant defences of thyrocytes [54–57].

#### Iron

Iron is an essential trace element for the function of all cells as it is crucial for energy metabolism and oxygen transport. Beyond its primary function, iron is required also for thyroid hormone synthesis because the thyroperoxidase enzyme (TPO) needs to bind with a heme group to become active on the apical membrane of thyroid cells. Moreover, iron is part of the prosthetic group of catalases, key antioxidant enzymes that counteract oxidative stress by catalyzing the reduction and detoxification of hydrogen peroxide. Therefore, iron deficiency, due to inadequate dietary intake or malabsorption, can impair thyroid hormone synthesis and secretion, increasing the risk of thyroid diseases [16, 54, 55]. Thus, frank and prolonged iron deficiency can favor the onset of hypothyroidism, while hypothyroidism is often associated with autoimmune gastritis or coeliac disease, negatively affecting iron absorption [16, 55]. Iron treatment prescribed to anemic women with hypothyroidism has been demonstrated to improve thyroid hormone levels, and the combination of thyroxine and iron proved more effective in improving iron status in this population group [58].

The average daily dietary iron intake is 10 to 15 mg in humans since only 1 to 2 mg is absorbed through the intestinal system. Dietary iron is present in two forms in food: heme and non-heme. Heme iron, derived from hemoglobin, is found in animal foods (fish, red meat and poultry). Non-heme iron is found in plant foods (nuts, whole grains, legumes, seeds, and green leafy vegetables), and is also the form of iron added to fortified and enriched foods. Heme iron is better absorbed than non-heme iron, and some dietary components can inhibit (phytates in whole grains, legumes, and nuts; tannins and polyphenols in coffee, tea, and red wine) or enhance (vitamin C) its absorption when consumed in the same meal [59]. Therefore, vegetarian/vegan diets are generally low in iron. Iron malabsorption can also be due to gastrointestinal disorders of autoimmune etiology (celiac disease, atrophic gastritis, IBD), which are often associated with autoimmune thyroid diseases [59, 60].

#### Zinc

Zinc, like iron, is involved in thyroid homeostasis, contributing to thyroid hormone synthesis and action at various levels: modulating TRH and TSH synthesis at the hypothalamic-pituitary level; regulating superoxide dismutase activity and thyroid hormone synthesis at the glandular level; regulating deiodinases (DIO) activity and T3 binding to its nuclear receptor in peripheral tissues. Moreover, zinc contributes to maintenance of oxidoreductive balance, and its deficiency can lead to increased oxidative stress [56, 57]. Since the body cannot store zinc, an adequate dietary zinc intake, estimated at 25 mg/day in adults, is recommended for maintaining normal thyroid function. Dietary sources of zinc include animal products (meat, poultry, and seafood), but whole grains and legumes can also be good sources of this trace element, considering that they also contain phytates that reduce absorption and bioavailability [61]. Consequently, zinc deficiency is common in vegans who exclude animal products, as well as in patients with nephropathies, malabsorptive gastrointestinal diseases, cancer and diabetes. However, while specific populations (such as subjects with documented zinc deficiency or at risk of severe malnutrition) appear to greatly benefit from zinc supplementation in terms of thyroid function, the role of zinc supplementation in the general population as a protective factor for thyroid disorders is still not fully established [62].

#### Selenium

Selenium is an essential cofactor for key enzymes in cellular metabolism, including antioxidant enzymes thioredoxin reductase (TrxR), glutathione peroxidase (GPx), iodothyronine deiodinases (DIOs), and selenoprotein P, all crucial for a correct thyroid function [63, 64]. Even small amounts of selenium are sufficient for correct DIO activity, but a reduced selenium intake affects GPx expression and function, leading to impaired antioxidant activity in thyroid cells. Therefore, selenium deficiency is associated with thyroid dysfunction and increased risk of thyroid autoimmunity and nodules [65], a condition further aggravated by the fact that concurrent iodine and selenium deficiency is rather common [66, 67].

Selenium can be found in foods in both organic (selenometionine and selenocysteine) and inorganic (selenate and selenite) forms. Major dietary sources include animal products like meat, seafood, eggs, nuts, and dairy products (including low-fat dairy). Plant-based foods like cereals and some vegetables and fruits also contain selenium, with amounts varying based on soil selenium content and other factors (including soil pH and the amount of organic matter in the soil) [55]. Selenium content of the soil can indirectly affect the meat content of selenium through the amount of selenium in the plants that animals eat. However, this does not give a relevant impact on the amount of selenium in animal products because animals maintain stable selenium tissue concentrations through homeostatic mechanisms [68]. Moreover, livestock feeds are generally enriched with selenium [69]. Lower selenium urinary concentrations were reported in vegetarians and vegans in some studies [70–73] although other studies showed similar levels between plant-based diets and omnivorous diets [74].

Although selenium deficiency, especially if severe, can impact thyroid health, it should be acknowledged that the role of selenium supplementation as a preventive or therapeutic strategy in thyroid diseases is highly controversial. Some studies suggest some benefit of adding selenium supplementation to conventional therapy in Graves’ disease (GD) [75, 76] and in Hashimoto’s thyroiditis (HT) [77, 78]. Nevertheless, other randomized clinical trials failed to demonstrate any beneficial effect of selenium supplementation in GD (81, 82) or in HT [79, 80]. Noteworthy, the European Thyroid Association (ETA) conducted an online survey among its members investigating the use of selenium supplementation across the spectrum of benign thyroid diseases. It emerged that, despite only a minority of responding ETA members stated that the available evidence warrants the use of Se in HT, the majority recommended it to some extent, especially to patients not yet receiving LT4 [81]. A recent meta-analysis suggested that Se supplementation could lower TSH and TPOAb levels, but only in patients with HT not treated with levothyroxine [82], reassessing the role of selenium in the management of patients with chronic thyroid disease [83]. The results of the clinical trials that have examined the effects of selenium supplementation on thyroid autoimmunity in pregnant women have also been controversial [84, 85], and the latest ATA guidelines recommends against selenium supplementation in pregnant women [86].

Selenium supplementation in euthyroid individuals without thyroid autoimmunity appears of negligible clinical relevance [87]. The only established clinical application of Selenium supplementation therapy is currently Thyroid Eye Disease (TED) [88–90]. The latest EUGOGO guidelines recommend a 6-month selenium supplementation for patients with mild and active TED of recent onset, because it improves eye manifestations and prevents progression to more severe forms [90]. In the general population, adequate dietary selenium intake is safe and beneficial for human health, with a recommended daily intake of 55 µg for adults, 0.2 µg/kg for children, and 65–75 µg for pregnant and lactating women [91]. However, selenium should not be routinely supplemented in non-deficient individuals [55, 63], in particular because chronic excess may lead to toxicity (selenosis). Related symptoms may include gastrointestinal upset, brittle nails, hair loss, and in more severe cases, neurological effects [92]. A Tolerable Upper Limit of 255 µg/day is established for adult men and women (including pregnant and lactating women) by the European Food Safety Authority (EFSA). Based on available intake data, adult consumers are unlikely to exceed the TUL, except for regular users of food supplements containing high daily doses of selenium [93].

#### Vitamins

Vitamin B12 is essential for a correct immune function [94]. Recently, its deficiency has been linked to an increased risk of thyroid autoimmunity since it impairs lymphocyte proliferation and antioxidant defenses. In AITD, borderline or low B12 levels are reported. A study involving hypothyroid patients showed that borderline-to-low B12 levels were more frequent in both overt and subclinical hypothyroidism groups compared with controls [95]. Another recent study examined vitamin B12 levels in patients with AITD and found them to be significantly lower compared to controls (*p* < 0.0001), with an inverse correlation to TPOAb levels [96].

All animal-based foods, such as fish, meat, eggs, dairy, and poultry, are good sources of vitamin B12 [97]. Consequently, vegetarian/vegan diets can cause a deficiency of this nutrient and require pharmacological supplementation. In addition to dietary intake, malabsorption conditions like celiac disease, atrophic gastritis, and inflammatory bowel diseases, all of autoimmune nature and frequently associated with autoimmune thyroid diseases, must be taken into account when vitamin B12 requirements are evaluated [97–99].

Vitamin D is a fat-soluble steroid with pleiotropic effects [100, 101]. In humans, vitamin D can be obtained from dietary sources or synthesized by sunlight-mediated skin synthesis. Cutaneous production of vitamin D is strongly influenced by anthropometric variables (body mass index, age, etc.), skin phototype, lifestyle and season [100, 101]. Few foods contain naturally significant amounts of vitamin D: fatty fish and cod liver oil are rich sources, whereas cream, butter, and egg yolk contain only small amounts, as do both cow’s and human milk. The use of pharmacological supplements and/or fortified foods is often necessary to ensure sufficient vitamin D levels [100].

Beyond its key role in skeletal homeostasis, several recent studies suggest extra-skeletal effects, such as a role as an immunomodulator in both adaptive and innate immune responses [102]. Observational clinical studies have shown that patients with autoimmune thyroid diseases have lower serum vitamin D levels than healthy controls [62, 103, 104]. Moreover, vitamin D deficiency has been correlated with higher titers of anti-thyroid autoantibodies, suggesting a role for vitamin D supplementation as an adjunctive tool for the therapy of thyroid autoimmune diseases [105]. However, intervention studies have shown only marginal effects of vitamin D supplementation on autoantibody levels without further clinical benefit [103, 106, 107]. Given the extensive and heterogeneous body of literature available—characterized by varying levels of evidence quality—readers are referred to dedicated, topic-specific reviews for more comprehensive insights and detailed analyses [108–110].

The data emerging from the most recent reviews and meta-analyses on vitamin D and vitamin B12 in relation to thyroid function and autoimmunity are summarized in Table 1. Overall, vitamins D and B12 are essential for general health, and their deficiency can impact thyroid health specifically, due to the resulting immune system dysregulation, favoring organ-specific autoimmunity in predisposed subjects. However, in the absence of demonstrated deficiency, pharmacological supplementation of these vitamins does not provide any demonstrated benefit in terms of prevention and/or treatment of autoimmune thyroid diseases [104, 106, 107, 110–118].

**Table 1:** Summary of major reviews and meta-analyses on vitamin D and vitamin B12 in relation to thyroid function and autoimmunity

Full reference	Study type	Population/sample	Main findings	Conclusions/remarks
Wang et al. [111]	Meta-analysis	20 studies; *n* = 3603 (1782 AITD; 1821 controls)	Patients with AITD had lower 25(OH)D (SMD − 0.99) and approximately threefold higher odds of deficiency (OR 2.99)	Strong association between vitamin D deficiency and AITD (Hashimoto’s and Graves’ disease)
Mazokopakis et al. [118]	Narrative review	Qualitative synthesis of 9 clinical studies	Vitamin D deficiency observed in 60–90% of patients with Hashimoto’s thyroiditis, associated with higher antibody titers	Suggests assessing and correcting vitamin D deficiency in autoimmune thyroiditis
Sun et al. [112]	Systematic review	> 25 clinical trials; > 3000 patients with Hashimoto’s thyroiditis	Vitamin D deficiency (< 20 ng/mL) associated with increased risk and higher anti-TPO; supplementation (2000–4000 IU/day) reduces antibody levels by 15–30%	Integrates mechanistic and clinical data; recommends supplementation only in documented deficiency
Tang et al. [113]	Meta-analysis	12 RCTs; *n* = 862	Vitamin D supplementation significantly reduced anti-TPO, anti-Tg, and TSH; greater effect with calcitriol and interventions > 12 weeks	Confirms duration-dependent and formulation-specific efficacy of vitamin D supplementation
Stefanić et al. [114]	Meta-analysis	35 observational studies; *n* > 6000	Lower 25(OH)D concentrations in Hashimoto’s thyroiditis (SMD − 0.94); age and latitude explain ~ 30% of variability	Reinforces the inverse relationship between vitamin D and thyroid autoimmunity; highlights geographical determinants
Jiang et al. [106]	Meta-analysis	6 clinical trials; *n* = 258	Vitamin D supplementation increased 25(OH)D and decreased anti-TPO (mean − 150 IU/mL); TSH remained unchanged	Quantifies dose–response; supports adjunctive role without clinical improvement
Taheriniya et al. [115]	Systematic review	42 observational studies; *n* ≈ 9500	Vitamin D deficiency doubles the risk of autoimmune thyroid disease (RR 2.3; 95% CI 1.7–3.1)	Provides broad epidemiological evidence linking vitamin D status to thyroid dysfunction
Benites-Zapata et al. [116]	Systematic review and meta-analysis	64 studies; *n* = 28,597 (24,835 hypothyroidism, 4901 AITD, 3795 hyperthyroidism)	Vitamin B12 levels lower in hypothyroidism (MD − 60.7 pg/mL); prevalence of deficiency 27% (hypo), 18% (AITD), 6% (hyper)	Highlights B12 deficiency in thyroid disorders; recommends routine screening in autoimmune hypothyroidism
Collins et al. [117]	Narrative overview	Review of 11 studies (~ 3000 patients)	Vitamin B12 deficiency prevalent (20–40%) in hypothyroidism and autoimmune thyroid disease	Early evidence summary linking thyroid disease and B12 deficiency; often cited in later meta-analyses

AIDT: autoimmune thyroid disease; TPOAb: anti-thyreoperoxidase antibodies; HT: Hashimoto’s thyroiditis

## Macronutrients and food components

Although the pathophysiological mechanisms underlying the relationship between food intake and thyroid are not fully understood, beyond the intake of certain micronutrients, it is possible to discuss the potential role of food components and macronutrients in thyroid homeostasis, with particular attention to autoimmunity.

### Polyunsaturated fatty acids (PUFA)

Polyunsaturated fatty acids (PUFA) are fatty acids characterized by a double bond along the carbon chain (the ω carbon) and are classified into two classes based on the position of the double bonds: ω-3 PUFA and ω-6 PUFA. Among these, alpha-linolenic acid (α-LA, an ω-3) and linoleic acid (LA, an ω-6) are defined as essential fatty acids since they cannot be synthesized in human body, and their intake fully depends on the diet [119]. ω-6 PUFA typically come from animal sources (meat, especially chicken, and eggs) and to a lesser extent from plant sources (soy, sunflower seeds and oils), while fatty fish and fish oils, leafy green vegetables, vegetable oils, nuts and seeds are rich in ω-3 PUFA [120]. Both ω-3 and ω-6 PUFA are essential components of phospholipid membranes and precursors of inflammatory mediators with opposing effects: ω-6 PUFA promote inflammation while ω-3 PUFA tend to inhibit it. Therefore, their health effects are strongly linked to dietary intake, especially their ratio. A balanced ω-6/ω-3 ratio is indeed important to support the anti-inflammatory profile of ω-3 PUFA, and a varied diet should include balanced amounts of each PUFA class with a predominance of ω-3. In fact, increasing evidence demonstrates the important role of PUFA and their bioactive metabolites (resolvins, protectins and maresins) in regulating inflammatory processes and protecting against and/or preventing degenerative, cardiovascular, and chronic inflammatory diseases, including autoimmune thyroid diseases [62, 119, 121, 122].

Given that fish is a primary dietary source of PUFAs, the impact of fish consumption on thyroid function and autoimmune processes is still a matter of debate. Indeed, the high risk of fish contamination by environmental pollutants cannot be ignored. In particular, while several studies suggest that ω-3 PUFA consumption has protective effects on the development of autoimmune thyroiditis [122], others demonstrate that exposure to pollutants (especially heavy metals) that contaminate fish is associated with an increased risk of autoimmunity and thyroid dysfunction [123–126]. This two-faced relationship highlights that fish consumption can be both beneficial and detrimental, with the ideal outcome depending on the type of fish, its origin (wild vs. farmed), and the presence and levels of contaminants [127]. However, the type of fish consumed, rather than fish consumption per se, plays an important role in assessing the cost (pollutants)/benefit (healthy components) ratio. Indeed, small oily fish (anchovies, mackerel, sardines), typical of the Mediterranean Sea catch, are a good source of PUFA and protein, and are associated with a low risk of heavy metal contamination. On the other hand, large predatory fish (such as swordfish) tend to concentrate pollutants, particularly persistent organic pollutants, heavy metals, and microplastics, due to biomagnification and bioaccumulation. Favoring the consumption of bluefish with low contaminant content can preserve the health benefits of fish while reducing the “toxic” effects [62, 127, 128]. Accordingly, in experimental models, supplements of omega-3 PUFAs have been demonstrated to exert neuro-protective and cardio-protective effects against hypothyroidism-induced damage, while attenuating hyperthyroidism-associated complications, including arrhythmias and hepatic dysfunction, thereby conferring protective actions on peripheral tissues across opposing states of thyroid dysfunction [129–132]. However, studies evaluating the role of omega-3 PUFA-based nutraceuticals in the setting of thyroid disorders, reached inconclusive results [133].

### Extra virgin olive oil (EVOO)

Extra virgin olive oil (EVOO), a vegetable fat extracted from the fruits of the *Olea europaea* plant, represents the primary source of dietary fat in the Mediterranean diet (MeD) and one of its most distinctive components [135]. It contains over 200 different bioactive compounds, including sterols, phenols, triterpenic alcohols and carotenoids. The phenolic composition of EVOO determines its strong antioxidant activity and is responsible for the pleiotropic positive effects of its consumption, ranging from metabolic and cardiovascular risk reduction to the improvement of chronic inflammation [134, 135]. In several experimental models of autoimmune diseases (including thyroiditis), EVOO components, oleic acid, hydroxytyrosol, oleuropein, and oleocanthal, have proven effective in counteracting inflammatory and oxidative imbalances, suggesting their efficacy in preventing autoimmune diseases [62, 136, 137].

### Gluten and cereals

Gluten is a mixture of storage proteins found in many cereals, the prolamins, which include glutenin and gliadin. Cereals, in turn, comprise a wide range of plant-based products derived from the Gramineae family, whose starchy seeds (caryopses) can be milled into flours used in human and animal nutrition. These include spelt, wheat, rye, oats, and barley, all containing gluten, and rice, corn, and millet, which do not contain gluten. There are also some so-called “pseudo-cereals,” not belonging to the Gramineae family, which include quinoa, buckwheat, and amaranth, all gluten-free [138]. Flours, cereals, and derived foods (pasta, bread, rice, ...) play a pivotal nutritional role as a source of carbohydrates, essential energy substrates for our body. Moreover, they provide small amounts of vitamins, proteins, and micronutrients and are, especially when whole, an important source of vegetable fibers, which contribute to the regulation of intestinal activity and microbiota. Finally, cereals contain a series of biologically active components (tocotrienols, folates, beta-glucans, fructans, lignans, polyphenols, phytosterols, policosanols) capable of exerting antioxidant and prebiotic activities, albeit in small quantities [138].

Gliadins, rich in glutamine and proline, and resistant to digestion by human proteases, are the trigger factor for celiac disease in genetically predisposed individuals [139]. For this reason, gluten is often stigmatized as responsible for alterations in human well-being and health [140]. As a consequence, the gluten-free diet, essential for individuals with celiac disease, has been proposed in recent years as a healthy dietary model also in non-coeliac individuals [141]. The supposed benefits of this diet would range from supporting digestive processes, to maintaining microbiota balance, contrasting overweight, and preventing inflammation and autoimmunity [140, 141]. Worth mentioning in this context is the so-called “immune-nutrition,” which aims to support the immune system’s function by eliminating dietary components such as lactose, gluten, and milk proteins (casein). These components are considered “immunological distractors” with pro-inflammatory activity, capable of causing dysbiosis and increased intestinal permeability (the so-called “leaky gut syndrome”), so favoring the development of autoimmune diseases [142]. Nevertheless, the current evidence remains largely inconclusive and does not demonstrate any clear benefit of a gluten-free diet (GFD) in patients without concomitant celiac disease, warranting further investigation [143–149]. Overall, there is currently not enough evidence to recommend a GFD to non-celiac patients with ATDs. Nevertheless, a GFD is increasingly recommended for individuals with autoimmune thyroiditis, exposing to the risk for unnecessarily worsening the quality of nutrition. Indeed, not only does the exclusion of many cereals from the diet reduce the nutritional intake of trace elements, proteins, and fibers, but gluten-free bakery products, often consumed as substitutes, are usually enriched with additives, fats, and sugars to improve palatability and texture [150].

### Dairy products

Milk and its derivatives are among the most studied products because of their potential effects in regulating pro-inflammatory and anti-inflammatory pathways in humans. Contradictory results in favor or against the pro-inflammatory effect of these products’ consumption in humans were published [151]. However, a systematic review, obtained analyzing 52 clinical trials, on the inflammatory markers related to the consumption of dairy products, underlined their prevalent anti-inflammatory activity [151], particularly evident for fermented products. Fermented foods were defined as *“foods made through desired microbial growth and enzymatic conversions of food components”* [152]. Several hypotheses were made about the health effects of fermented foods such as nutritive alteration of food ingredients and the biosynthesis of different compounds, variations of the gut microbiota, and the immune system modulation [152]. A recent paper demonstrated that the risk of developing Hashimoto’s thyroiditis in children of mothers who, during pregnancy, consumed homemade yogurt was significantly, even slightly, lower. A lower risk of developing this disorder was also observed in children who consumed fermented food as homemade yogurt and cheese [153].

On the other hand, a restriction in lactose intake is suggested in hypothyroid patients in need for levothyroxine treatment affected by lactose malabsorption, a condition with a global prevalence of 68%, due to the non-persistence of the expression of lactase gene [154]. The undigested lactose molecules may attract an increased amount of fluids in the bowel lumen, leading to accelerated intestinal transit and possible reduction of absorbed levothyroxine [155].

In conclusion, diets “without” (lactose, gluten, …), in the absence of proven intolerance, do not improve overall human health, nor thyroid health. Instead, they might deprive the individual of important nutrients, and can even lead to eating disorders, the so-called “orthorexia,” a kind of obsession with “healthy eating” that leads to an arbitrary elimination of every food deemed harmful to the diet.

### Cruciferous vegetables

Cruciferous vegetables belong to the Brassicaceae family that include cauliflower, kale, turnips, broccoli, Brussels sprouts and others that are rich in glucosinolates. During mastication they are enzimatically converted into thiocyanates, goitrin metabolites, that cause a competitive inhibition of the sodium/iodide symporter, thus inhibiting the thyroid hormones’ synthesis and possibly causing hypothyroidism [156]. These effects, described in animal models, were not generally observed in humans. A single case report described the development of myxedema coma in a patient accustomed to consuming for months large amounts of raw Chinese white cabbage (such as bok choy) [157]. The anti-thyroid (goitrogenic) activity of Chinese white cabbage is primarily attributable to the release of the enzyme myrosinase following tissue disruption, such as during mastication or raw processing. Myrosinase catalyzes the hydrolysis of endogenous glucosinolates into biologically active goitrogenic compounds, including thiocyanates and nitriles, potentially resulting in goiter and hypothyroidism. Notably, myrosinase is heat-labile, and this process is therefore effectively inhibited by cooking [157]. Aside from this single case report, a RCT on the effect of a broccoli sprouts extract administration in 45 patients for 84 days demonstrated no variations in TSH, FT4, anti-TPO and anti-Tg autoantibodies positivity rate [158]. Another study demonstrated in 5 patients that the ingestion of 430 g of kale juice leads to an increase in serum and urinary thiocyanate concentrations and to a significant decrease in the 6-hour thyroid ^123^I uptake while the 24-hour thyroid ^123^I uptake was not affected by its intake. Moreover, serum TSH and FT4 remained unchanged [159]. The authors concluded that although in the short-term kale juice ingestion does not lead to thyroid dysfunction, adequate iodine intake should be recommended in subjects ingesting routinely large amounts of cruciferous vegetables. Even the cooking method should be considered, since it may impact on the amount of goitrogen compounds released [160].

### Soy based-products

Animal studies demonstrated the TPO-inhibiting activity of soy-based products because of their content in isoflavons (genistein, daidzein and glycitein). Since the early sixties it was described the occurrence of goiter and hypothyroidism in children fed soy-based formula [161]. Indeed, in patients with subclinical hypothyroidism or iodine deficiency, soy isoflavonoids ingestion may lead to clinically relevant effects [161]. However, a recent systematic review carried out in 2017 revealed that euthyroid iodine-repleted subjects exposed to soy isoflavonoids have a slight TSH increase with no changes in FT3 and FT4 levels [162]. To note, Pinchera et al. [163], described the case of an athyreotic cretin who showed a refractoriness to high doses of desiccated thyroid when fed soybean formula due to an increased fecal concentration of ^131^I-labeled levothyroxine, compared with data obtained from the same child when on a whole milk diet, thus suggesting a possible levothyroxine malabsorption. A similar effect on levothyroxine treatment efficacy was also described in a thyroidectomized patient taking soy protein supplement near to the ingestion of her levothyroxine daily dose [164]. An appropriate separation between the ingestion of levothyroxine and soy protein-based compounds should be advised to warrant a proper absorption of the hormone [154].

## Different dietary models

Several studies have evaluated the association between dietary models and the development of thyroid diseases in Western societies, suggesting that moderate consumption of animal products and a predominant intake of plant-based foods may be protective against thyroid diseases, especially autoimmune ones [13–16, 34, 62, 165].

The main clinical studies addressing the relationship between dietary habits and thyroid functional status and/or autoimmunity are summarized in Table 2. In early 2010s’, Tonstad and coworkers investigated the prevalence of thyroid dysfunction in members of the Seventh-day Adventist church, a population largely following plant-based diets. They reported that vegans had a lower risk of both hypo- and hyperthyroidism compared to omnivores, supporting a protective role of plant-based diets. More in detail, prevalence of hyperthyroidism (independently from its origin) was higher in omnivores compared to vegans, vegetarians and lacto-ovo vegetarians [166], whereas the prevalence of hypothyroidism did not differ significantly between the different dietary patterns’ groups despite a tendency to reduced risk in vegetarians [167].

**Table 2:** Summary of the clinical studies addressing the relationship between dietary habits and thyroid functional status and/or autoimmunity

Study	Dietary habits	Study design	Thyroid effects
Tonstad et al. [167]	Vegetarian diet vs. Omnivorous diet	Observational clinical study on 65,981 subjects, members of the Seventh-day Adventist Church	Reduced risk of prevalent (OR 0.89, 95% CI: 0.78–1.01) and incident (OR 0.78, 95% CI: 0.59–1.03) hypothyroidism in vegetarians
Tonstad et al. [166]	Vegetarian diet vs. Omnivorous diet	Observational clinical study on 65,981 subjects, members of the Seventh-day Adventist Church	Lower risk of hyperthyroidism in vegan diets (OR = 0.49; 95% CI 0.33–0.72), lacto-ovo-vegetarian diets (OR = 0.65; 95% CI 0.53–0.81), and pesco-vegetarian diets (OR = 0.74; 95% CI 0.56–1.00) compared to omnivorous diets.
Liu et al. [168]	Dietary Inflammatory Potential (DIP) score	Cross-sectional study including 2346 U.S. male subjects aged ≥ 20 years (data from NHANES)	Positive association with serum TT4 levels (β = 0.07; *p* = 0.0044); no effect on fT3, fT4, or TSH levels
Chen et al. [169]	Dietary Inflammatory Index (DII)	Cross-sectional study involving 964 HT patients (67.6% females). Mean age: 51.4 ± 16.2 years	Positive correlation between DII and TSH and total T4 levels
Klobučar et al. [170]	Dietary Inflammatory Index (DII)	Cross–Sectional multicenter study involving 149 adults diagnosed with HT (140 females), aged 19 to 72 years	DII was positively correlated with TSH (*p* = 0.002) and BMI (*p* = 0.04)
Zupo et al. [171]	Adherence to the Mediterranean Diet (PREDIMED score) – Extra Virgin Olive Oil (EVOO) consumption	Observational study on a cohort of 324 overweight/obese euthyroid subjects (228 women and 96 men, aged 14–72 years)	Inverse relationship between PREDIMED score and serum levels of fT3 (*p* < 0.01) and fT4 (*p* < 0.01); no effect on serum TSH levels
Giannakou et al. [172]	Frequency of consumption of food groups normal vs. low fruit consumption and daily vs. sporadic vegetable consumption	Observational study including 218 euthyroid HT women aged 46.0 ± 12.7 years	Low fruit and vegetable consumption and high BMI were associated with high TOS
Alijani et al. [173]	Dietary Inflammatory Index (DII) and dietary total antioxidant capacity (DTAC)	Hospital-based case–control study involving 115 HT patients (54.5% females) and 115 controls ( 5.5% females) Mean age 39.76 ± 9.52 years	In the HT group, the DII level was higher (*p* < 0.001) and the DTAC level was lower than those in the healthy group (*p* = 0.047) DII positively correlated with TPOAb, TGAb and TSH levels, while DTAC had a negative correlation with anti-TPO and TG-Ab (*p* < 0.050)
Kaličanin et al. [174]	Frequency of consumption of food groups	Observational study including 491 patients with Hashimoto’s thyroiditis and 433 controls	Higher consumption of animal fats (OR = 1.55, *p* < 0.0001) and processed meat (OR = 1.16, *p* = 0.0012) in patients with Hashimoto’s thyroiditis Higher consumption of red meat (OR = 0.80, *p* < 0.0001), soft drinks (OR = 0.82, *p* < 0.0001), whole grains (OR = 0.82, *p* < 0.0001), and vegetable oil (OR = 0.87, *p* < 0.0001) in controls Association between vegetable oil consumption and increased fT3 levels in patients with Hashimoto’s thyroiditis (β = 0.07, *p* < 0.0001)
Ruggeri et al. [175]	Frequency of consumption of food groups – Adherence to the Mediterranean Diet (PREDIMED score)	Observational study including 81 patients with Hashimoto’s thyroiditis (71 women, 10 men) and 119 controls (102 women, 17 men)	Higher frequency of animal-based food consumption (meat, *p* = 0.0001; fish, *p* = 0.0001; dairy products, *p* = 0.004) in patients with Hashimoto’s thyroiditis Higher frequency of plant-based food consumption (legumes, *p* = 0.001; fruits and vegetables, *p* = 0.030; nuts, *p* = 0.0005) in controls Lower adherence to the Mediterranean diet in patients with Hashimoto’s thyroiditis compared to controls (*p* = 0.0001). The PREDIMED score is an independent predictor of TPOAb positivity (OR = 0.192; 95% CI 0.074–0.500; *p* = 0.001)
Corrias et al. [176]	Adherence to the Mediterranean Diet (PREDIMED score)	16 euthyroid patients (96 F, aged 57.2 ± 13.1 years) affected by thyroid disorders (70%, autoimmune thyroid disease), 248 healthy adults (65% F, aged 53.1 ± 12.1), from Sardinia, Italy	Lower adherence to the MD (*p* = 0.003) was reported in patients compared to healthy subjects
Candussi et al. [177]	Comparison between several dietary groups (vegans, vegetarians, pescatarians, poultry-eaters, low meat-eaters, and high meat-eaters)	Registry study, evaluating data from the UK Biobank, concerning eating habits of 466,362 individuals aged 40–69 years	Higher risk of hypothyroidism among vegetarians compared to people following a high meat diet, mostly after correction for BMI (HR = 1.23, 95% CI 1.07–1.42). Positive association between low meat-eaters, poultry-eaters, pescatarians and vegetarian with hypothyroidism prevalence
Kim et al. [178]	Comparison between different dietary patterns: the plant-based diet (PBD), the Western-style diet (WSD), and the balanced Korean diet	Hospital-based cohort of 56,664 participants aged > 40	Subjects in the high-PBD, low-WSD, and smoker groups had a higher proportion of hypothyroidism than those in the low-PBD, high-WSD, and non-smoker groups

TSH: thyroid stimulating hormone or thyrotropin; TT4: total thyroxine; fT4: free thyroxine; fT3: free triiodothyronine; TFTs: thyroid function tests; TPOAb: anti-thyreoperoxidase antibodies; HT: Hashimoto’s thyroiditis; MD: Mediterranean Diet; PREDIMED: PREvención con DIeta MEDiterránea; MDS: Mediterranean Diet Score; FFQ: food frequency questionnaire; DTAC: dietary total antioxidant capacity; DII: dietary inflammatory index; BMI: body mass index; EVOO: extra-virgin olive oil; NHANES: National Health and Nutrition Examination Survey; TOS: total lipid peroxide levels

Since then, several studies have explored the influence of dietary patterns on thyroid function and autoimmunity. Some focused on individual nutrients, while others considered the overall inflammatory potential of the diet, measured through indices such as the Dietary Inflammatory Index (DII). Using NHANES data, Liu et al. found that higher DII scores were associated with increased TT4 and TT3 levels—still within the normal range—without significant changes in fT3, fT4, or TSH [168]. Chen et al., also using NHANES, observed that HT patients following more pro-inflammatory diets showed higher TSH and TT4, though no significant association with thyroid antibodies was found [169]. Similarly, Klobučar et al. reported that HT patients adhering to anti-inflammatory diets had lower TSH, higher fT4, and reduced BMI, supporting a possible link between dietary inflammation and thyroid hormone balance [170]. On the other hand, a greater adherence to the MeD —particularly higher consumption of EVOO—was associated with lower fT3 and fT4, while no association was found with TSH, in overweight and obese euthyroid individuals [171].

Other studies addressed diet and thyroid autoimmunity. Giannakou et al. demonstrated that daily fruit and vegetable consumption contributes in maintaining oxidative stress at low levels in HT patients [172]. Alijani et al. found that pro-inflammatory diets correlated with higher TSH and antibody levels, while greater dietary antioxidant capacity was linked to reduced autoantibodies. Frequent intake of fruits and vegetables also appeared to lower oxidative stress in HT patients [173]. Kaličanin et al. reported that HT patients consumed more animal fats and processed meat, whereas controls ate more whole grains, vegetable oils, and unprocessed red meat. Patients on levothyroxine therapy also consumed more red meat than untreated patients. Importantly, dietary habits often remain unchanged after diagnosis, suggesting that nutritional management is underappreciated in clinical care [174]. Ruggeri et al. demonstrated that HT patients had higher consumption of animal-based foods and sweetened processed products, while controls favored vegetables, legumes, and nuts. MeD adherence was significantly lower among patients, and the PREDIMED score independently predicted thyroid antibody positivity, indicating a protective role of the MeD [175]. During the COVID-19 pandemic, individuals with thyroid diseases (mostly autoimmune) showed lower adherence to the MeD, higher prevalence of overweight, and reduced physical activity compared with controls, further supporting the benefits of healthy lifestyle choices [176].

Recently a population-based prospective study on 466,362 individuals from the UK Biobank’s hospital inpatient data [177] showed that none of the types of diet evaluated (high and low meat-eater, poultry-eater, pescatarian, vegetarian and vegan) was significantly associated with the risk of hypothyroidism in models without adjustment for BMI. After adjusting for BMI, the association for vegetarians (HR = 1.23) resulted statistically significant. This result was in keeping with the one reported in a recently published paper [178]. The authors made different hypotheses to explain this data: 1) the lower iodine intake, frequently detected in vegetarians and vegans compared to other type of dietary pattern users, as well as 2) the possible role of the lower iron intake that characterizes subjects following a plant-based diet [177].

Although findings in the literature are sometimes conflicting and overall inconclusive, evidence suggests that a predominantly plant-based diet—rich in antioxidants, fiber, and micronutrients—may be advisable over dietary patterns characterized by excessive consumption of animal products, particularly animal fats and red meat, provided that adequate intake of iodine and other essential micronutrients is ensured. Two contrasting dietary models warrant consideration: the western-style and the MeD diet.

Worth of note, the two dietary models differently impact the microbiota composition. In the last years, a link between gut microbiota composition and autoimmune diseases has been proven in murine models and hypothesized in humans owed to the interactions with both the innate and adaptive branches of immunity [179]. In this view, the imbalance between commensals and pathogens (namely dysbiosis) would enhance the entry of bacterial and food antigens into the systemic circulation, triggering auto-aggressive processes [180]. The action might also be mediated by variations in Short Chain Fatty Acids (SCFA), bacterial byproducts known to exert robust antinflammatory activity [181]. In contrast, bacterial lipopolysaccaride (LPS) is able to trigger thyroiditis in NOD H2h4 mice, by interacting with TLR4 expressed on thyrocytes. Studies on gut microbiota transplantation in animals confirmed that dysbiosis may increase thyroid autoimmunity susceptibility [179]. To note, the main bacterial species that are associated with gut barrier leakiness, such as Bacteroides fragilis and Fusobacterium, were increased in patients with AITD. On the other hand, protective bacterial species, such as Bifidobacterium, Lactobacillus and Butyricicoccus, that promote a healthy gut barrier and enhance immune tolerance, are reduced in patients with HT [182].

### Western diet

In recent decades, a diet high in fats (mainly saturated and trans fatty acids and cholesterol), calories, and proteins (mainly meat), rich in refined sugars and salt, and low in fiber has spread in Westernized countries. This dietary model has been indicated as the “Western diet” (WD) and is associated with overnutrition and an increased risk of obesity and related comorbidities [183, 184]. A distinctive feature of the WD is the excessive consumption of both ultra-processed and fast-foods, rich in unhealthy components typical of the WD, such as saturated fats and refined carbohydrates, but poor in healthy nutrients, such as vitamins, fibers, and trace elements. Moreover, these foods have a high content of additives (emulsifiers, colorings, sweeteners, preservatives) and other “industrial” ingredients (such as hydrolyzed proteins, hydrogenated or de-esterified oils), used to make the final product more palatable [185]. WD habits promote a state of chronic metabolic inflammation (the so-called “metainflammation”) both directly due to the pro-inflammatory effects of their components and indirectly through overweight/obesity and increased fat mass [184]. Furthermore, the low consumption of vegetables and fruits causes a lack of exogenous antioxidants, while an excessive intake of animal proteins (mainly red and processed meat) and fats has been associated with increased oxidants, increasing the risk of immune-mediated disorders, including autoimmune thyroid diseases [186, 187]. Moreover, the WD model creates an unfavorable environment in the gut and alters the functions of the intestinal barrier and microbiota composition, leading to dysbiosis and inflammation [188]. Altered intestinal permeability (“leaky gut”) allows the passage of food antigens, toxins, bacteria, and microbial products, promoting inflammation and dysregulated immune responses, triggering autoimmunity through various mechanisms [188].

### Mediterranean diet

The Mediterranean diet (MeD) is a nutritional life style which has been developed and inspired by the dietary traditions of populations living in the Mediterranean Sea area, olive-growing lands, and is characterized by a high consumption of plant-based foods (fruits, cereals, vegetables, nuts, legumes, seeds, and olives), fish and seafood, and moderate amounts of dairy products, meat, and meat products, and of red wine during meals. EVOO is the main source of lipids and represents a high-quality nutritional food due to its richness in bioactive compounds, mainly phenols [15, 62, 121, 186]. The positive ratio of ω-6/ω-3 PUFA, vitamins (E, B, C, and β-carotene), fibers, minerals (selenium, iron, zinc, iodine) are key components of the MeD and account for its beneficial effects [15, 62, 121, 186]. Moreover, the MeD positively influences the composition and health of gut microbiota, also supporting the functional and anatomical integrity of the intestinal barrier and proper immune stimulation [189].

**Fig. 2:**
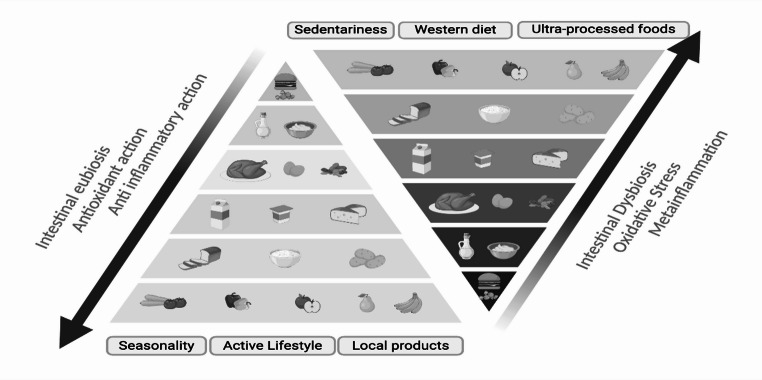
Contrasting effects of Mediterranean and Western dietary patterns on thyroid health. The Mediterranean diet, rich in plant-based foods, extra virgin olive oil, and antioxidants, supports gut eubiosis, modulates inflammation and exerts protective effects on thyroid autoimmunity. Conversely, the Western diet, characterized by high intake of ultra-processed foods, saturated fats, and refined sugars, promotes dysbiosis, oxidative stress and systemic metainflammation, which may contribute to thyroid dysfunction and autoimmune activation

Several clinical studies have shown a positive effect of MeD on the reduction of the risk of developing chronic metabolic diseases such as diabetes mellitus, cardiovascular diseases, arterial hypertension, but also cancer, neurodegenerative diseases and osteoporosis. It is important to note that the positive health impact of the MeD derives from the synergy of bioactive compounds and nutrients that interact with each other, producing increasing beneficial effects, which cannot be replicated by pharmacologically supplementing a single component [15, 62, 175, 190]. The MeD represents a balanced and complete dietary model, favoring plant-based foods, such as vegetables, whole grains, and EVOO, without excluding animal products, thus ensuring an adequate intake of vitamins and micro- and macronutrients essential for thyroid function. For its demonstrated antioxidant, immunomodulatory, and anti-inflammatory effects, the MeD plays a protective role against chronic autoimmune and inflammatory diseases [15, 62]. Therefore, the MeD represents a healthy dietary model to recommend to patients with thyroid diseases, in contrast to the WD (Fig. 2).

### Autoimmune protocol diet

From the perspective of inflammation as the trigger of thyroid autoimmunity, several different “elimination” diets were proposed in the attempt to avoid foods that are considered pro-inflammatory. The autoimmune protocol diet (AIP) is considered as an evolution of the Paleolithic diet since it is personalized based on patients’ symptoms [191]. This is characterized by an elimination phase, lasting 6 weeks to 6 months, in which grains, legumes, nuts seeds, dairy, eggs, coffee and alcohol are forbidden. The second phase consists in the gradual reintroduction of eliminated foods identifying the ones that may trigger symptoms. The third phase is the maintenance one. Two studies evaluated the effect of this dietary approach on HT patients. In the first one [192], 16 patients followed AIP for 10 weeks: no variations of thyroid function test or autoantibodies levels were observed after the AIP diet. The second one [193] evaluated thyroid hormones and volume variations in 28 patients before and after 12 weeks of this dietary regimen: both fT3 and fT4 were significantly reduced, as well as thyroid volume, while TSH values were only slightly lower than before the AIP period. On the contrary, anti-TPO antibodies were slightly increased, without reaching statistical significance.

While both studies demonstrated enhanced emotional and physical well-being and a reduction in body weight and BMI, significant alterations in thyroid function tests were observed only in the study by Ihnatowicz et al. [193] seemingly reflecting an adaptive response to the energy deficit rather than a direct dietary effect on thyroid function.

### Low carbohydrate diet

A few papers evaluated the effect of low-carbohydrate diet on thyroid functional and immunological status, involving mostly obese patients. Obesity represents itself as a challenge for hypothalamus-pituitary-thyroid axis since it has been hypothesized that hyperleptinemia, that often characterizes these patients, may increase the expression of TRH gene and thus TSH secretion [194]. In turn, this causes an increase in thyroid volume. One recent paper [195] examined the effect of low-carbohydrate diet on thyroid inflammation in HT patients through MRI semi-quantitative assessment of the water content of the thyroid. After six months, the 20 HT patients who followed a low-carbohydrate diet showed a significant reduction of thyroid water content as well as decrease of both anti-TPO and anti-Tg autoantibodies compared to HT patients who had followed a normal diet [195]. Unfortunately, the small sample size and the lack of thyroid function data of enrolled patients prevent drawing conclusions on this possible effect. Further studies are needed to confirm the effect on thyroid homeostasis of low carbohydrate diet.

## Conclusive remarks

In conclusion, despite its promise, nutrition remains an underrepresented focus in clinical research, underscoring the need for robust, large-scale, evidence-based investigations. In this context, maintaining overall health and supporting optimal thyroid and immune function depend on a varied and balanced diet, as no single nutrient or pharmacological supplement can ensure complete nutritional adequacy or sustain physiological homeostasis. It is therefore essential to follow varied nutrition to optimize thyroid homeostasis and minimize the risks of developing immune alterations and thyroid dysfunctions.

Understanding the influence of dietary habits on autoimmune disorders has become increasingly critical, as the global prevalence of these conditions continues to rise and curative treatments remain limited. While genetic and environmental determinants are well recognized, diet constitutes a modifiable factor capable of shaping immune regulation, inflammatory pathways, and gut microbiota composition—central mechanisms in autoimmune disease pathogenesis. Particular attention to dietary quality is warranted, especially in light of emerging and re-emerging nutritional deficiencies that may compromise immune function and exacerbate disease susceptibility. Investigating dietary patterns, such as adherence to anti-inflammatory or Mediterranean-type diets, as well as the adequacy of specific micronutrient intakes, may provide valuable insights into preventive strategies, symptom modulation, and improved patient outcomes. Given the dramatic increase in autoimmune disease prevalence and the associated social and healthcare burden, integrating nutritional approaches into prevention and management frameworks may represent a cost-effective and powerful tool in addressing this expanding global health challenge.

## Declarations

### Animal study

The authors of this article did not conduct any animal studies.
